# Disaggregating physiological components of cortisol output: A novel approach to cortisol analysis in a clinical sample – A proof-of-principle study

**DOI:** 10.1016/j.ynstr.2019.100153

**Published:** 2019-03-07

**Authors:** Veronika B. Dobler, Sharon A.S. Neufeld, Paul F. Fletcher, Jesus Perez, Naresh Subramaniam, Christoph Teufel, Ian M. Goodyer

**Affiliations:** a Department of Psychiatry, Developmental Psychiatry, University of Cambridge, 18b Trumpington Rd, Cambridge, CB2 8AH, UK; b Cambridgeshire and Peterborough NHS Foundation Trust, Elisabeth House, Cambridge, CB21 5EF, UK; c Norwich Medical School, University of East Anglia, Norwich, NR4 7TJ, UK; d Department of Neuroscience, Instituto de Investigacion Biomedica de Salamanca (IBSAL), University of Salamanca, Spain; e Cardiff University Brain Research Imaging Centre, School of Psychology, Cardiff University, Maindy Road, Cardiff, CF24 4HQ, UK

**Keywords:** Childhood adversity, Psychopathology, Cortisol, Stress-induction, Waking cortisol, Diurnal variation

## Abstract

Although childhood adversity (CA) increases risk for subsequent mental illnesses, developmental mechanisms underpinning this association remain unclear. The hypothalamic-pituitary-adrenal axis (HPAA) is one candidate system potentially linking CA with psychopathology. However, determining developmental effects of CA on HPAA output and differentiating these from effects of current illness has proven difficult. Different aspects of HPAA output are governed by differentiable physiological mechanisms. Disaggregating HPAA output according to its biological components (baseline tonic cortisol, background diurnal variation, phasic stress response) may improve precision of associations with CA and/or psychopathology. In a novel proof-of-principle investigation we test whether different predictors, CA (distal risk factor) and current depressive symptoms, show distinct associations with dissociable HPAA components. A clinical group (aged 16–25) at high-risk for developing severe psychopathology (n = 20) were compared to age and sex matched healthy controls (n = 21). Cortisol was measured at waking (x4), following stress induction (x8), and during a time-environment-matched non-stress condition. Using piecewise multilevel modeling, stress responses were disaggregated into increase and decrease, while controlling for waking cortisol, background diurnal output and confounding variables. Elevated waking cortisol was specifically associated with higher CA scores. Higher non-stress cortisol was specifically associated with higher depressive scores. Following stress induction, depressive symptoms attenuated cortisol increase, whilst CA attenuated cortisol decrease. The results support a differential HPAA dysregulation hypothesis where physiologically dissociable components of HPAA output are differentially associated with distal (CA) or proximal (depressive symptoms) predictors. This proof-of-principle study demonstrates that future cortisol analyses need to disaggregate biologically independent mechanisms of HPAA output.

## Highlights

• Update of cortisol analysis.
• Disaggregation of biological components of HPA axis output.
• Associations of early adversity and/or illness are tested for each component.
• Early adversity relates to high waking cortisol and attenuated post-stress recovery.
• Current symptoms relate to high daytime cortisol and less adaptive cortisol release.

## 1 Introduction

Many mental disorders, including depressions and psychoses are associated with exposure to childhood adversities (CA). One potential pathophysiological pathway from CA to symptoms may involve long-term alterations of the Hypothalamic-pituitary-adrenal axis (HPAA) function leading to measurable differences in steroid outputs including cortisol, adrenocortiocotropic hormone (ACTH) and corticotropin releasing hormone (CRH) (Herbert, 2013; McEwen, 1998; Wilkinson and Goodyer, 2011). Both human and rodent literature suggest that mechanisms modulating cortisol output are in part influenced by early rearing factors during infant and childhood periods supporting programming effects enduring through to adult life (Meaney et al., 2002; Taylor et al., 2010; Weaver et al., 2004; Wilkinson and Goodyer, 2011). Attempts to correlate variations in HPAA outputs to CA and psychopathology have however yielded inconsistent findings within and across psychiatric diagnostic categories (Ciufolini et al., 2014; Fogelman and Canli, 2018; Young et al., 2000). This has made the formation of a robust developmentally sensitive theory of HPAA dysregulation for subsequent mental disorders rather problematic. Alterations in HPAA output may also be a consequence of dynamic endocrine effects that change with disease state independent of any effects of CA - but this has yet to be established.

The last few decades have continued to reveal increasingly complex biological mechanisms underpinning HPAA function (Karatsoreos and McEwen, 2011; McEwen, 2007; Sousa et al., 2008). In general terms, HPAA output can be differentiated into tonic (background) and phasic (reactive) components. Tonic cortisol release is also subject to diurnal variation regulated by the central circadian clock, a function of the hypothalamic suprachiasmatic nucleus (Czeisler et al., 1980; Dickmeis, 2009). In contrast, short-term phasic HPAA activation, such as seen when confronted by novel, unexpected or uncontrollable stimuli (Dickerson and Kemeny, 2004), is governed via various neural mechanisms involving the prefrontal cortex, limbic and brain stem regions including activation in the Locus ceruleus, eventually resulting in cortisol secretion. Phasic cortisol response to acute stress has multiple effects on subsequent neurophysiological adaptation (allostasis), including the release of energy and shifts of cognitive network activation (Danese and McEwen, 2012; Hermans et al., 2011).

In humans, with sleep being a period of relatively low differentiation to environmental influences, waking cortisol is considered as marker of baseline tonic output (Bartels et al., 2003; Kupper et al., 2005). Support for elevated waking cortisol as trait-like biomarker predicting depression has been demonstrated in adolescents (Owens et al., 2014). While specific mediation through CA exposure was not firmly established, heritable mechanisms partly accounted for these findings (Bart et al., 2006; Schreiber et al., 2006).

Further, a number of studies have suggested that CA may be associated with individual differences in stress related cortisol output (Calhoun et al., 2014; MacMillan et al., 2009). Compromise of phasic cortisol output (Wilkinson and Goodyer, 2011) may be a physiological signature of ineffective adaptive coping (allostatic failure) to current life stressors (Danese and McEwen, 2012; de Kloet et al., 2005). However, findings have been equivocal with CA predicting both increased (Elzinga et al., 2003; Heim et al., 2002) and decreased (Calhoun et al., 2014; Carpenter et al., 2007; Elzinga et al., 2008) cortisol output under experimental stress in mentally ill participants.

Acute cortisol response to stress consists of two components: i) initial increase, determined by a cascade of physiological mechanisms, including CRH, ACTH and cortisol secretion, and ii) termination of the response (cortisol decrease) which relies on efficient negative feedback through glucocorticoid (GR) receptors. Both components (increase and decrease) are sensitive to the impact of CA, for example, via interaction of early rearing factors with vulnerability genes leading to long-term alterations in the control of HPAA output (Mahon et al., 2013; Tyrka et al., 2009; Zannas and Binder, 2014). For instance, CA has been associated with i) attenuated post-stress cortisol decrease via epigenetic alterations of GR receptor sensitivity (Zannas and Binder, 2014), ii) greater post-stress cortisol increase due to epigenetic influences on CRH activation (Tyrka et al., 2009). These effects are considered as independent of each other and illustrate two distinct mechanisms accounting for altered post-stress cortisol levels in vulnerable individuals.

In summary, components of cortisol output are underpinned by differentiable control mechanisms: background diurnal output subserved by the suprachiasmatic nucleus and the circadian clock; reactive increase in cortisol, subserved by a neurally sensitive cascade system via CRH activation; baseline tonic cortisol levels and post-stress cortisol restitution controlled via negative feedback at GR receptors. To date the impact of either CA and/or current illness on these components has not been systematically differentiated, thus possibly accounting for inconsistent findings.

We conjecture that to better understand the impact of CA on HPAA function in patients with mental illnesses requires measuring the following HPAA outputs in a single experimental design: tonic waking cortisol (baseline output), diurnal background cortisol (non-stress), and both components of phasic cortisol (increase and decrease) in time-environment-matched non-stress and stress conditions. To undertake this strategy a multivariate modeling procedure is required that allows for piecewise disaggregation of cortisol components when testing for specific associations between different cortisol outputs and CA and/or current symptoms.

Based on these considerations we speculate that a history of CA would be associated with alterations in HPAA components where programming effects are predicted. In contrast, we consider that current symptoms are more likely associated with an overall general elevation in daytime cortisol levels. We therefore test the following hypotheses:

1. Greater CA will be associated with elevated waking cortisol levels and attenuated post-stress cortisol decrease due to programing effects impairing negative feedback mechanisms (Zannas and Binder, 2014). Higher waking cortisol likely correlates with impaired post-stress physiological recovery.
2. Higher symptom scores will be associated with elevated cortisol levels across the non-stress condition due to illness related effects such as distress (Dienes et al., 2013).
3. CA may predict greater post-stress cortisol increase in a subgroup, due to programing effects on CRH release (Tyrka et al., 2009).

In the present study we used a well-validated experimental stress induction paradigm (Kirschbaum et al., 1993) measuring post-stress phasic cortisol outputs in a late adolescent/young adult (aged 16–25) clinical group at high risk of developing severe psychopathology versus age and sex matched controls with no lifetime history of mental illness. As cortisol release shows high diurnal variability with different rates of decline throughout the day and also responds to a wide range of internal or external triggers, we measured diurnal variation (without induced stress) under equivalent environmental conditions at time-matched diurnal times. In addition, we established baseline tonic cortisol levels at waking by measuring morning waking cortisol over four days prior to experimental procedures. Current symptoms and CA were ascertained via questionnaires. We applied updated analytical approaches (see below) to partition out the contribution of baseline cortisol and diurnal variation, as well as taking account of intra-individual and inter-individual variations of sampling, and timing of peak cortisol concentrations. This allows assessing distinct associations of different cortisol outputs under waking, non-stress and stressful conditions with CA and current symptoms in patients.

## 2 Materials and methods

We conducted a case-control experiment comparing a Clinical Group (CG) with community ascertained Healthy Controls (HC). Three HPAA output components were measured: 1) morning waking cortisol, 2) cortisol following stress induction, 3) daytime (diurnal) cortisol in a non-stress condition. The latter two were measured on separate days at equivalent time-points (details below). The study was approved by the NRES East of England committee and was performed in accordance with relevant guidance and regulations.

### 2.1 Participants

CG (n = 20) were recruited from a specialist mental health service for young people aged 16–35 years, presenting for the first time with psychotic symptoms, meeting criteria for at-risk mental state for psychosis according to the Comprehensive Assessment of At Risk Mental States (CAARMS) (Yung et al., 2003), but not for a full psychotic episode (Supplementary Information: Full criteria for At-risk mental states). HC (n = 21) were recruited from a panel of volunteers with no lifetime history of mental illness. All participants took part in this study as part of a project investigating the relationship between CA, changes in HPAA output, alterations in cognitive performance and symptoms in young people at-risk of developing severe psychopathology. Herein we report on the characterization of the HPAA profile of the participants.

Psychotic experiences (such as hallucinations) have a relatively high prevalence in adolescent/young adult populations (Varghese et al., 2011; Wigman et al., 2012) (Kelleher et al., 2012a), but have been found to represent an unspecific symptomatic marker of increased risk for developing severe psychopathology with high levels of co-morbidity across the spectrum of anxiety, depression and psychotic disorders (Kelleher et al., 2012b). Psychotic experiences are also strongly associated with a history of CA (Caspi, 2010; Kelleher et al., 2013, 2008). Our clinical group therefore represented individuals with a high-risk for developing severe psychopathology and a high probability of having experienced CA (Varghese et al., 2011; Wigman et al., 2012). Particularily in young populations, symptoms fluctuate and tend to dynamically develop across various diagnostic categories over time (Caspi et al., 2014; Cramer et al., 2010). Equally, some mood or anxiety related symptoms may be experienced in healthy populations without reaching pathological significance or impairment. Recent research suggests that, especially in young populations with emerging mental illness, a dimensional approach may be better suited to adequately describe current psychopathology (St Clair et al., 2017; Stochl et al., 2015). In the present study, current symptoms/clinical status were therefore characterized in four ways; i) determining at-risk status (CAARMS), ii) determining the current formal categorical DSM IV diagnosis via a clinician-led MINI current mental state diagnostic interview (Sheehan et al., 1998), iii) determining symptoms dimensionally in relation to depression, delusional thought content, and anxiety proneness on continuous scales and, iii) determining level of functioning/impairment. Exclusion criteria included habitual smoking, current use of antipsychotic medication, steroid medication or contraceptive pill (Supplementary Information: Full recruitment procedure, inclusion/exclusion criteria). General cognitive functioning (IQ) was established using the Catell culture fair test of intelligence (Cattell, 1940).

### 2.2 Procedures

Participants were telephone screened for eligibility and, if they met criteria, invited for an initial interview and written informed consent prior to participation in the study. Participants were given instructions for at-home saliva collection and asked to bring the samples to the testing sessions. All testing followed a fixed protocol and timing whereby all procedures were conducted at the same times of day. Stress and non-stress days were counterbalanced according to a pre-set matrix. Dates were set a minimum of 2 weeks apart. Prior to each testing session participants were screened for compliance with the inclusion criteria. Stress induction was followed by end-of-session debriefing.

### 2.3 Cortisol samples

#### 2.3.1 Waking cortisol

Patients were instructed to collect saliva upon waking on two days prior to coming to each testing session (4 samples in total). A kit with Salivettes (SalimetriCG^®^) and full written instructions were provided (Supplementary Information: Written instructions).

#### 2.3.2 Test days salivary cortisol collection

Samples were collected at set time points between 12.45 and 14.30 in the laboratory on each testing day (TSST: eight sessions; non-stress: six sessions). The stress condition consisted of a well validated stress induction procedure (Trier Social Stress Task (TSST) (Kirschbaum et al., 1993)). The TSST is a standardized socially evaluative stress induction, which includes elements of anticipation (participants are introduced to a panel of judges and asked to prepare a presentation for a job interview), public speaking (presentation in front of panel) and mental arithmetic (in front of panel). Stress induction elicited “TSST cortisol”. The non-stress condition assessed background diurnal daytime variation at the time of testing, under controlled laboratory conditions (“non-stress cortisol”). To control for mental and physical activity, participants were asked to follow instructions of a progressive muscle relaxation tape of equal duration to the TSST. Baseline cortisol was collected prior to either intervention. All other samples were collected post-intervention (stress/non-stress) at as close as possible matching time points for both conditions. On both days participants performed the same set of computer tasks and questionnaires post-intervention whilst collecting salivary samples. (Supplementary Information: Fig. S1).

The saliva samples were stored in a freezer upon receipt and analyzed at the local Core Biochemical Assay Laboratory (CBAL) (Cambridge University Hospitals NHS Foundation Trust), using SalimetriCG^®^ Salivary Cortisol Enzyme Immunoassay Kit for duplicate cortisol analysis.

All cortisol data were logged in order to minimize the impact of outliers (Hruschka et al., 2005).

### 2.4 Symptom scales and measures for CA

Current mood symptoms in the whole sample were established with the self-reported Beck Depression Inventory BDI (Beck et al., 1996) immediately prior to the stress and non-stress sessions, with the mean score being used in analyses. The Peters Delusion Inventory (PDI) (Peters et al., 1999) was obtained as a measure of abnormal beliefs (appropriate for clinical and non-clinical populations) and the State-Trait-Anxiety Inventory (STAI-T) (Spielberger, 2010) was used to ascertain levels of anxiety-proneness. Both were obtained once at the beginning of the study, as was the level of everyday functioning using the Global Assessment of Functioning (GAF, DSM-IV). CA was assessed using the self-reported Childhood Trauma Questionnaire (CTQ) (Bernstein et al., 2003). Of the symptom measures, BDI has the greatest established clinical validity (Storch et al., 2004). Given that it also was assessed immediately before each testing session, we chose the BDI as the most proximal index of symptoms when examining associations between current symptoms and HPAA components.

### 2.5 Data analysis

The aims of the data analysis were threefold: first to establish waking cortisol characteristics; second to reveal characteristics of the diurnal cortisol in the non-stress condition; third to characterize the effects of stress on increase and decrease of cortisol levels taking both waking cortisol levels and non-stress (diurnal) levels into account.

Multi-level mixed-effects linear regression using maximum likelihood was performed to interrogate the panel cortisol data. Prior studies assessing cortisol reactivity have utilized this approach as it accounts for intra-individual variation of baseline (Hruschka et al., 2005), missing data, unequally spaced data points (Willett et al., 1998), and non–independence of repeated measures data (Hruschka et al., 2005). A piecewise approach has previously been taken to separate cortisol reactivity and recovery post-stress, also with a similar sample size to the present study (Hollocks et al., 2014).

However, here we also account for both waking cortisol and non-stress cortisol, and use individual peak cortisol levels instead of group means.

Primary outcomes were waking cortisol, TSST (stress)- and non-stress cortisol. The effects of our three key predictors, clinical group (primary predictor), BDI (proximal predictor), and CTQ (distal predictor), were assessed in separate models due to high correlations among the predictors. In order to effectively test the association of BDI and CTQ with cortisol, we pooled HC and CG to allow for a more complete range of BDI and CTQ scores, in models where these were predictors. In each model, IQ, age, test day order, gender, and waking cortisol (for TSST and non-stress cortisol outcomes) were assessed as fixed-effect confounders; those which were related to both the outcome (p < 0.1) and predictor (p〈0.1 or r/ρ〉 = 0.1) were included in each model. Thus, not all confounders will be the same in each model. Repeated measurements were nested within individuals (a random-effect). In models predicting waking cortisol, main fixed-effects of predictors were assessed, along with fixed-effects of day and time of waking, and random-effects of time of waking, due to repeated assessments across four days. For TSST and non-stress cortisol outcomes, time of measurement was modeled as a fixed (linear and quadratic) and random-effect (linear only), as participants' cortisol measurements were not always equally spaced in time (Supplementary Information: Table S1). Key predictors (HC/CG, BDI, CTQ) were included in separate models, each as a fixed interaction with time to assess the influence of these factors on cortisol's change over time (i.e.: slope). Putative confounders which correlated with these predictors were also independently interacted with time on cortisol; those interactions which were p < 0.01 were included in each relevant model. Despite a small sample size, we were able to include confounders in the models, as all our models contained well over two subjects per variable, the requirement necessary in regression models for adequate estimation of regression coefficients, standard errors, and confidence intervals (Austin and Steyerberg, 2015).

*TSST baseline, peak, and end of the cortisol reaction:* The value used for TSST baseline cortisol response was obtained immediately prior to the start of the TSST. This was an average of 20 min (SD = 4) after participants arrived in the testing room, allowing relaxation after the physical exertion of getting to the appointment. As the timing of peak cortisol response varies between individuals (Kirschbaum et al., 1993), we assessed which of three theoretically plausible peaks reflected each participant's maximum cortisol output. These peaks were set to be the highest TSST cortisol value at a mean of 23 min (SD = 1), 29 min (SD = 2), or 36 min (SD = 2) after the start of the task (74). The end of the TSST cortisol reaction was set at a mean of 58 min (SD = 5) after the start of the task, a mean of 22 (SD = 2) to 34 (SD = 5) minutes after the peak. To separately model cortisol reactivity increase and decrease, the dataset was split into two at the TSST peak cortisol level (varying by individual), allowing for piecewise analysis of these phases.

*TSST Extreme responders:* Given prior reports suggesting that CA is related to overall decreased post-stress cortisol but increased cortisol release in a subgroup (Tyrka et al., 2009), we explored the sample for potential extreme responders prior to further analysis. TSST cortisol slope from start to peak was calculated and inspected for outliers.

*TSST minus non-stress cortisol*: In order to consider TSST cortisol reactivity whilst controlling for individual variation in cortisol in the same environment under non-stress condition (“non-stress cortisol”), we subtracted logged non-stress cortisol from logged TSST cortisol. Prior to this, we accounted for individual variation in the timing of sample collection by imputing the non-stress data to the exact time points of the TSST data (Supplementary Information: Fig. S2). Piecewise multilevel modeling described above were repeated with “TSST minus non-stress cortisol” as the outcome.

## 3 Results

### 3.1 Participant characteristics

**Fig. 1:**
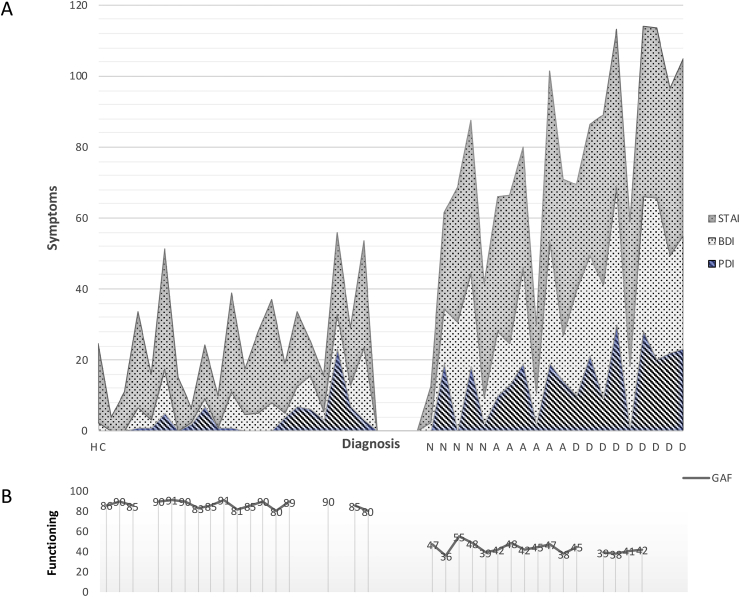
Symptom characteristics (A) and impairment (B) within diagnostic categories in Clinical Group (CG) and Healthy Controls (HC). A: Cumulative scores in self-rated questionnaires on delusional thought content (PDI), anxiety proness (STAI-T) and depression (mean BDI) in HC and CG (N = no diagnosis, A = anxiety spectrum, D = depression). While some sypmptoms were present in the HC with no lifetime history of mental illness, in the CG all symptom scales were significantly elevated. B: In comparison to the HC the CG was functionally impared according to the GAF.

CG (n = 20; 14 male) had a mean age of 20.8 years (SD = 2.75) and HC (n = 21; 11 males) had a mean age of 20.2 (SD = 3.25). While all patients were referred due to psychotic symptoms, 75% (n = 15) met criteria for a diagnosis based on the MINI diagnositic interview (Sheehan et al., 1998). Most common primary diagnoses were depressive diagnoses (n = 8 (40%)), followed by anxiety spectrum diagnoses (n = 7 (35%)). As expected in this age group, 60% (n = 12) also had at least one secondary diagnosis (7 depressive, 5 anxiety); three met criteria for a third diagnosis. Despite being identified as at-risk-mental-state, 5 participants did not meet full criteria for a diagnosis according to the MINI, although they exhibited a sufficient range of symptoms and/or impairment in functioning to justify inclusion in the clinical group (Fig. 1; Supplementary Information: Table S2). Eight patients took psychotropic medication, usually SSRIs (Supplementary Information: Table S2). As expected, the CG versus HC showed significantly higher symptoms, CA, and impairment (Fig. 1A and Table 1). CG/HC was collinear with BDI and CTQ (rho 0.87 and 0.84 respectively). There was no significant between-group difference in IQ (Table 1). Across both groups, in the full dataset, CTQ scores were strongly correlated with BDI scores (r = 0.63), and BDI was colinear with PDI and STAI (r = 0.81 and r = 0.85 respectively). Among those in the clinical group, there were increased levels of trait anxiety (STAI-T), psychotic believes (PDI), and depressive symptoms (BDI) irrespective of diagnosis. Further, all diagnostic groups showed a mean score of depressive symptoms (BDI) above clinical threshold (mild depression). Two HC reported below threshold unusual perceptual experiences/unusual thought content on the CAARMS.^3^ There was one HC who exceeded the clinical cut-off for depression on the BDI (20), one high outlier on the PDI, and 10 HC with self-reported increased levels (>35) of anxiety proness. However these were without associated functional impairment, therefore not meeting criteria for a clinical diagnosis. Further, there were 5 HC who had experienced moderate or severe CA based on CTQ, and 7 CG who had experienced none or low CA, reflecting a range of experiences across the samples. *In summary*: although some mild symptoms were reported in our HC, the CG and HC differed significantly on all symptom levels, CA and impairment.

**Table 1:** Participant characteristics.

	Combined mean (SD)	n	HC mean (SD)	n	CG mean (SD)	n	*t*-test	p
BDI^a^	14.43 (13.15)	41	5.13 (5.21)	21	24.20 (11.81)	20	−6.75	<0.0001
PDI	8.78 (9.20)	40	3.38 (5.19)	21	14.74 (9.05)	19	−4.93	<0.0001
CTQ	41.88 (17.98)	40	31.05 (4.41)	20	52.70 (19.93)	20	−4.74	<0.0001
STAI-T	47.71 (14.28)	41	37.52 (8.85)	21	58.40 (10.58)	20	−6.87	<0.0001
GAF	65.55 (22.40)	33	86.53 (3.91)	17	43.25 (4.97)	16	27.89	<0.0001
IQ	117.29 (17.80)	41	121.33 (18.11)	21	113.05 (16.87)	20	1.51	0.14

HC=healthy controls; CG=clinical group; BDI=Beck Depression Inventory; PDI=Peters Delusion Inventory; STAI-T=State-Trait-Anxiety Inventory (Trait Anxiety); GAF=Global Assessment of Functioning; IQ=General cognitive functioning.

a Mean of scores taken prior to each testing day.

### 3.2 TSST exclusions

One HC was excluded from all TSST analyses due to consistently high and improbable cortisol levels throughout the TSST (>3 dl, (Salimetrics ^®^ Assay range: 0.012. −3.000 μg/dl, with 3 dl representing the ceiling of the test). However, this participant's data was retained in any tonic (morning and non-stress) cortisol analysis as these levels were well within range of other HCs. Three CG had extreme responses on the TSST, with mean slope 6.5 times greater than all the remaining participants (M = 0.88, SD = 0.28; Supplementary Information: Fig. S3), including the HC, and therefore would be inappropriate to include. As the extreme responders were clearly too few to analyze separately, they were excluded from any TSST analyses, bringing the sample to 17 CG and 20 HC. (These individuals' non-stress cortisol data was normal and therefore was used in non-stress analyses). Additionally, one individual's cortisol did not increase during the TSST; these data were used for cortisol increase, but cortisol decrease was not calculated due to no prior increase.

### 3.3 Waking cortisol

**Fig. 2:**
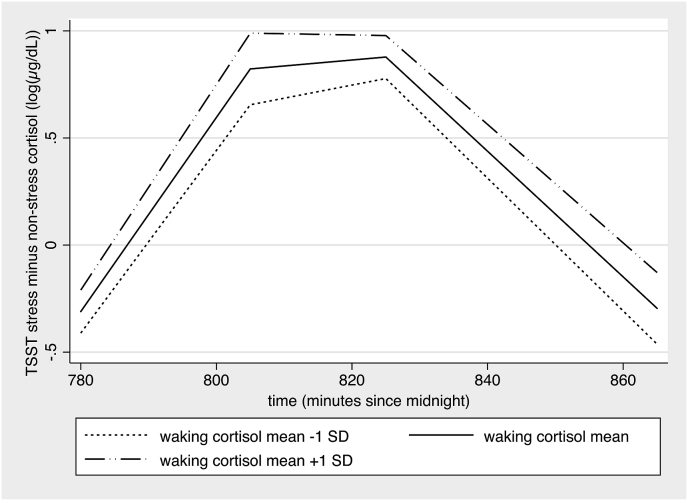
Predictive margins^b^ of waking cortisol on “TSST minus non-stress cortisol” increase and decrease (TSST = Trier Social Stress Test), adjusted by confounds. Piecewise analysis of cases and controls together revealed that waking cortisol levels did not influence “TSST minus non-stress cortisol” increase (Coef = 0.32 × 10^−2^ (95% CI: 0.73 × 10^−2^, 1.36 × 10^−2^), p = 0.55; confound: gender x time) or decrease (Coef = 0.49 × 10^−2^ (−0.54 × 10^−2^, 1.53 × 10^−2^), p = 0.35; confounds: age x time, gender). However, waking cortisol had a positive influence on overall “TSST minus non-stress cortisol” levels, non-significant from TSST start to peak (Coef = 0.21 (−0.02, 0.43), p = 0.074; confound: gender), but significant from TSST peak to finish (Coef = 0.34 (0.03, 0.65), p = 0.031; confound: gender). *Sample size:* Four TSST ouliers were excluded (see method and results). Waking cortisol was missing from one participant. The decline phase additionally excluded one participant who exhibited no cortisol increase from the TSST. All TSST slope increase and decrease models have 3 to 5 timepoints per person, which varies depending on when each individual peak occurred. This resulted in n = 132 (36 participants) for analyses of cortisol increase, and n = 151 (35 participants) for cortisol decrease.

Waking cortisol was not significantly different between the four sampling days, nor did time of waking influence cortisol as a fixed-effect. However, inclusion of time of waking as a random-effect improved model fit (Akaike information criterion decreased) and thus was included in all models with waking cortisol as outcome. One HC participant was missing waking cortisol data. Only CTQ was positively associated with waking cortisol; no association was found with HC/CG or depressive symptoms (Table 2). Waking cortisol was also assessed as a predictor of non-stress and “TSST minus non-stress cortisol””, with confounds included as described in methods. Waking cortisol did not influence non-stress cortisol decline (i.e.: no interaction with time: linear p = 0.59, quadratic p = 0.49) or non-stress cortisol levels across the whole testing period (p = 0.79). Additionally, waking cortisol did not influence slope increase or decrease in the “TSST minus non-stress” condition, but it did yield a positive influence on overall cortisol values from TSST peak to finish (Fig. 2).

**Table 2:** Waking cortisol.

Distal and proximal influences on waking cortisol^a^				
Effect	N^b^	Coef	95% CI	*p*
Main Effects				
Day	151 (39)	−4.87 × 10^−2^	−13.42 × 10^−2^ to 3.68 × 10^−2^	0.26
Time of waking	151 (39)	−0.07 × 10^−2^	−0.29 × 10^−2^ to 0.15 × 10^−2^	0.52
HC/CG (1 = CG)	143 (37)	6.37 × 10^−2^	−26.02 × 10^−2^ to 38.75 × 10^−2^	0.70
CTQ	147 (38)	0.92 × 10^−2^	0.36 × 10^−2^ to 1.49 × 10^−2^	0.001
BDI	151 (39)	0.42 × 10^−2^	−1.24 × 10^−2^ to 2.08 × 10^−2^	0.62

Waking cortisol levels were not influenced by HC/CG, but they were associated with increased CTQ scores.

a Multilevel model across 4 days. Only the CTQ model required adjustment for confounding (gender was included). All models include random effects of time of waking (missing in 1 individual), but findings remain when this random effect is not included.

b Bracketed number refers to the number of cases. Waking cortisol was missing from one HC participant, and time of waking was missing for another. CTQ was missing from an additional participant. HC/CG analyses excluded two participants who were outliers on BDI or PDI.

### 3.4 Cortisol under non-stress condition

**Fig. 3:**
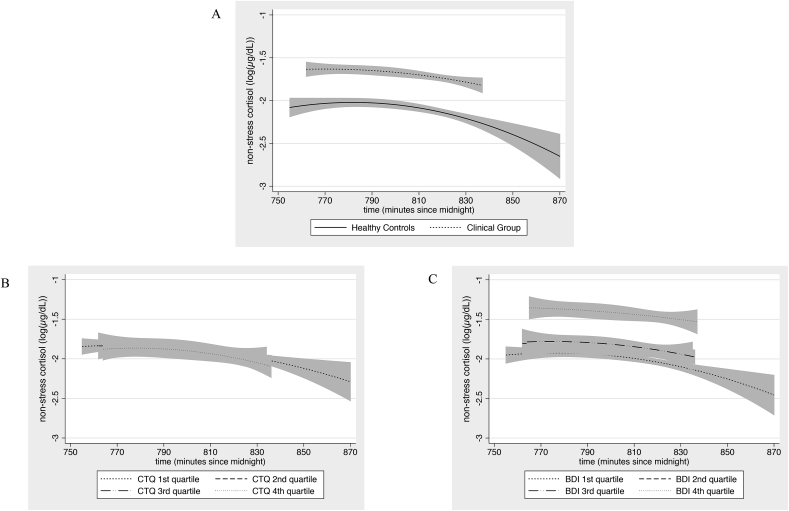
Influence of predictors on non-stress cortisol, adjusted by confounds. Predictors did not influence non-stress cortisol slope (all interactions with time and time2, p ≥ 0.36). Therefore main effects of predictors are presented, adjusted for confounding, and including the quadratic effect of time. 95% Confidence intervals are from the standard error of prediction. Continuous predictors were divided into quartiles to depict their influence on non-stress cortisol. A) The clinical group (CG) exhibited higher levels of non-stress cortisol than the healthy controls (HC) throughout the testing period (Coef = 0.47, (0.19, 0.75), p = 0.001; confound: gender; n = 236 [41 participants x 6 timepoints.]). B) Across the whole sample, CTQ scores did not influence non-stress cortisol levels (Coef = 0.34 × 10^−2^ (−0.45 × 10^−2^, 1.13 × 10^−2^), p = 0.40); confounds: gender, IQ; n = 240 [40 participants x 6 timepoints. One participant was missing CTQ]). C) Across the whole sample, those with higher BDI scores had higher levels of non-stress cortisol throughout the testing period (Coef = 1.11 × 10^−2^ (0.33 × 10^−2^, 1.89 × 10^−2^), p = 0.005; confounds: IQ, test day order; n = 246 [41 participants x 6 timepoints]).

There was a significant diurnal decline of cortisol over time (linearly: Coef = −0.26 × 10^−2^ (−0.49 × 10^−2^ to −0.02 × 10^−2^), p = 0.033; quadratically: Coef = −6.25 × 10^−5^ (−11.90 × 10^−5^ to −0.60 × 10^−5^), p = 0.030). None of the predictors influenced this slope. CG versus HC and BDI scores were positively associated with higher overall cortisol across all time points (Fig. 3).

### 3.5 Cortisol and TSST condition

**Fig. 4:**
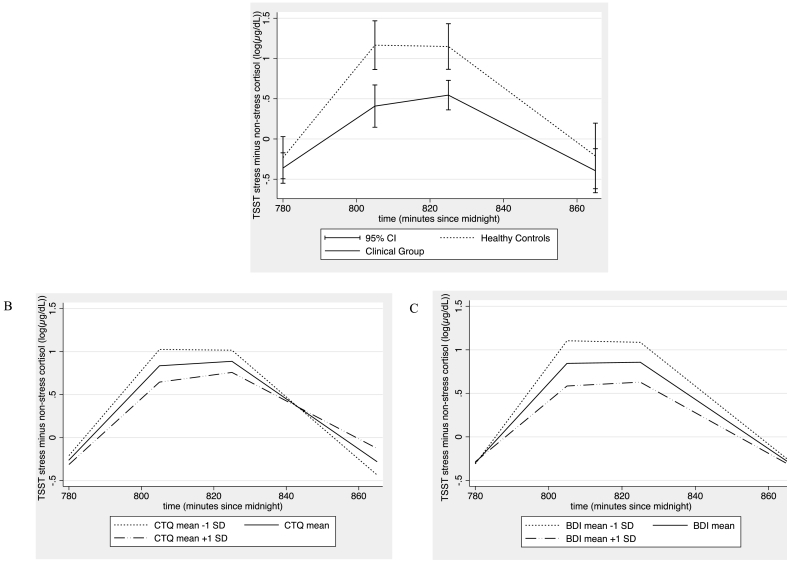
Influence of predictors on “TSST minus non-stress cortisol” increase and decrease, adjusted by confounds. Predictive margins^b^ are used in B and C to show the influence of a continuous predictor on cortisol slope. A) The clinical group (CG) exhibited an attenuated increase (Coef = −10.57 × 10^−3^ (95% CI: 19.92 × 10^−3^, −1.21 × 10^−3^), p = 0.027) and trend level decrease (Coef = 9.56 × 10^−3^ (1.27 × 10^−3^, 20.38 × 10^−3^), p = 0.084) in cortisol compared with healthy controls (HC), as shown by significant interactions of HC/CG with time. B) Across the whole sample, CTQ scores did not influence cortisol increase (p = 0.51; full statistics presented in Table S3) but higher CTQ scores attenuated cortisol decrease (Coef = 0.31 × 10^−3^ (0.03 × 10^−3^, 0.59 × 10^−3^), p = 0.028). C) In the whole sample, higher BDI scores attenuated cortisol increase (Coef = −0.41 × 10^−3^ (−0.76 × 10^−3^, −0.05 × 10^−3^), p = 0.025) but not decrease (p = 0.15). (See Table S3 for confounds and sample size; TSST = Trier Social Stress Test). ^b^For depicting effects of continuous predictors (CTQ and BDI) on “TSST minus non-stress cortisol”, predictive margins were computed from each model at the mean of the predictor, and ± 1SD from the mean. (Predictive margins are computed probabilies of the outcome at specified values for the independent variable in the model.

Piecewise analysis of “TSST minus non-stress cortisol” revealed linear but not quadratic effects of time in the increase (baseline to peak) and decrease (peak to end) phase (Supplemental Information: Table S3) Therefore only interactions with time (not time2) were assessed. An attenuated cortisol increase was noted in CG compared with HC, which was a trend level for cortisol decrease (Fig. 4A). Higher BDI scores attenuated cortisol increase (Fig. 4C), and higher CTQ scores attenuated cortisol decrease (Fig. 4B). Findings with “TSST minus non-stress cortisol” differed from TSST uncontrolled for diurnal cortisol. TSST cortisol uncontrolled by non-stress cortisol showed CG, CTQ, and BDI had no effect on cortisol increase but all attenuated cortisol decrease (Supplementary Information: Table S4).

In summary, a differential pattern of predictors of the differential aspects of cortisol output emerges:

i) Higher CTQ scores were associated with elevated morning cortisol levels.
ii) BDI scores were associated with overall elevated cortisol in the non-stress condition.
iii) In the stress condition (controlling for the non-stress cortisol levels) current depressive symptoms were associated with attenuated cortisol increase. Conversely, higher CTQ scores were associated with attenuated cortisol decrease.

## 4 Discussion

While both HPAA and CA have been related to various types of psychopathology, correlations within and across psychiatric diagnostic categories have been inconsistent (Ciufolini et al., 2014; Fogelman and Canli, 2018; Young et al., 2000). Recently it has been suggested that new approaches may be nessecary to tackle heterogeneity in findings of HPAA outputs in relation to CA and psychopatology (Fogelman and Canli, 2018). Within the present study, we set out to systematically disaggregate the associations between current depressive symptoms and self-reported CA with components of HPAA output in participants at-risk to developing severe psychopathology (CG) and HC with no lifetime history of mental illness. We chose this strategy because of potentially distinct underlying latent mechanisms governing waking cortisol levels, diurnal decline and components of phasic cortisol response (increase and decrease) that may be differentially susceptible to exposure to CA and/or current symptoms. We hypothesized that CA and current illness would show different associations with distinct components of HPAA output. In order to test this, we needed to disaggregate the phasic response to stress into its physiological components of increase and subsequent decrease, distinguish high from low responders, control for baseline tonic output at waking and partition out the alterations in non-stress diurnal cortisol output at the same time of day.

Consistent with our first hypothesis, we revealed that elevated tonic waking cortisol was associated specifically with higher CTQ scores regardless of group (case-control). Furthermore, higher CTQ scores were also specifically associated with attenuated phasic post-stress cortisol *decrease*. Elevated waking cortisol correlated with higher post-peak cortisol after stress induction.

Given the small sample size and lack of genetic data to control for genetic effects on morning waking cortisol levels or post-stress decrease, we cannot make definite claims regarding the precise mechanism underpinning these effects. However, CA related, epigenetically determined downregulation in GR sensitivity, with successive impairments in negative feedback mechanisms may provide one possible explanation for the association between CA, elevated waking cortisol as marker of increased baseline tonic output, and attenuated post-stress recovery, independent of symptom related effects. Our findings are theoretically consistent with developmental programming effects following CA exposure, which only affect distinct components in HPAA output in vulnerable populations, but are potentially related to long term risk of successive severe psychopathology. If confirmed in future studies this provides a potential explanatory framework for morning waking cortisol acting as a biomarker amongst the adolescent ‘at risk’ population for major depression (Owens et al., 2014), that is explained by both heritable mechanisms and CA.

In contrast, cortisol levels measured during the non-stress condition were positively associated only with symptom scores of current depression, suggesting that, the more severe the illness profile the greater the likelihood of an overall elevated diurnal cortisol output. The results are consistent with our second hypothesis and support an independent effect of current symptoms on parameters of HPAA output, irrespective of CA. These latter results resonate with studies noting that a proportion of currently depressed patients show a reversible loss of day and nighttime diurnal rhythm in their HPAA function, and this may be more likely in the most severely ill patients (Binder et al., 2004). To our knowledge, this is the first study specifically assessing diurnal variation at time-matched intervals to the stress related phasic response. The above findings strongly support our methodological approach of controlling for non-stress characteristics of the HPAA output in order to systematically disaggregate the impacts of CA and current symptoms respectively when investigating HPAA phasic response to stress in patients.

In addition, we noted that greater depressive symptoms correlated with attenuated post-stress phasic cortisol *increase*. We hypothesized that perhaps during illness, already increased daytime diurnal HPAA output may limit the physiological reserve for adaptive (allostatic) post-stress cortisol increase.

Previous clinical studies have suggested both an increased and attenuated phasic cortisol response to acute stress in populations with CA and mental illness (Calhoun et al., 2014; Carpenter et al., 2007; Elzinga et al., 2008, 2003; Heim et al., 2002). In line with our third hypothesis we identified a small subgroup of clinical participants showing extreme cortisol reactivity. The theoretical approach to analyses in this study is guided by understanding of the biological underpinnings of cortisol realease to date. On this basis, excluding these from the rest of the sample was justified: based on previous literature, they likely represent a further physiologically distinct clinical subgroup related to gene-by-environment mechanisms affecting CRH control (Bradley et al., 2008; Tyrka et al., 2009). Future studies with larger sample sizes might be able to further explore CA/illness related associations in such subgroups.

To date findings of HPAA abnormalities have been inconsistent within or across diagnostic categories. Co-morbidity in DSM IV diagnoses was common in our sample. This was expected as the CG was recruited based on psychotic symptoms, as marker for high-risk of emerging severe psychopathology (Kelleher et al., 2012b) and related to high levels of co-morbidity in young populations. Therefore, and in recognition of increasing calls for dimensional definitions of mental health in research (Caspi et al., 2014; Krueger et al., 1998) we chose a dimensional approach to characterizing current psychopathology across several symptom domains of depression, anxiety and abnormal beliefs. This also allowed pooling data across samples for some of the analyses. Despite the categorical diagnostic hereogeneity within the CG, higher levels of symptoms in all domains were seen in CG versus HC, irrespective of CG diagnosis. The symptom profile of our group resonates with the increasing recognition that diagnostic categories represent less distinct pathologies than previously thought, but overlapping symptom clusters (Cramer et al., 2010), with a potentially common latent factor underpinning mood, anxiety and psychotic disorders (Stochl et al., 2015). CA and HPAA abnormalities are associated with many mental illnesses. CA related programming effects on physiological changes may be present irrespective of the dynamic effects of current symptom status (Faravelli et al., 2010), and possibly represent a latent mechanism common to these symptom clusters.

Stress induction in clinical populations is notoriously difficult to conduct due to primary and secondary contraindications of inflicting stress upon an unwell population. Therefore most current data rely on sub-clinical population studies or small numbers in clinical groups (Heim et al., 2002; Hollocks et al., 2014; Young et al., 2000). Clearly, conclusions of this study are limited by its small sample size and replication is needed in larger sample sizes using similar methodology. For example, larger sample sizes would allow for assessment of cortisol profiles separately by clinical status and/or gender. However, to our knowledge this is the first study employing piecewise multilevel modeling to fully disaggregate different physiological components of the phasic HPAA response in mentally ill patients. By using this approach, we maximize the power of a small data set over 2 experimental days (stress and non-stress). We also provide a proof-of-principle for the importance of this statistical disaggregation by revealing putatively different effects on tonic, diurnal and phasic cortisol outputs and further demonstrate the importance of controlling for the non-stress diurnal levels in such populations.

Theoretically, distinguishing physiological subgroups is of value in preparation for delineating developmental pathways resulting from the impact of CA. For example, CA has been theorized to evoke specific changes in HPAA function, and putative subsequent downstream developmental events (Cicchetti, 2010), with specific influence on neurocognitive processes (Diamond et al., 2007; Lupien et al., 2007). Indeed, high waking cortisol levels have been associated with overgeneralized autobiographic memories (Owens et al., 2014). Future developmental theories of HPAA dysregulation in the pathogenesis of mental disorders could benefit from accounting for the heterogeneity of cortisol profiles (Fogelman and Canli, 2018; Hagan et al., 2015).

## 5 Conclusions

Herein we present a novel approach to cortisol analysis which systematically disaggregates components of HPAA output. Such analysis has allowed us to distinguish effects of CA and current depressive symptoms on cortisol levels in young people at high-risk for developing severe mental illness. Our findings provide preliminary novel evidence for a differential HPAA axis dysregulation hypothesis regarding the impact of CA and current depressive symptoms on specific components of HPAA output. We suggest that the elevated waking cortisol and attenuated cortisol decrease post-stress in this population likely reflects negative glucocorticoid feedback as a result of programing effects via an interaction of CA with vulnerability genes, rather than being directly symptom related. Impaired negative GR feedback has been associated with various disorders, including depression and psychcosis (Zannas and Binder, 2014). Consistent with the clinical presentation of our sample (multiple, mixed symptoms, high prevalence of CA) our sample might represent a subgroup of youth at-risk of a more severe course of illness, greater treatment resistance, and lifelong risk of for recurrent illness. Future studies will need to establish the programing effect of CA vesus later life adversity. During illness, elevated daytime cortisol output may deplete cortisol reserves and further compromise the capacity for adaptive physiological responsiveness to current stress, and thus contribute to maintenance of symptom levels. These conclusions are clearly tentative due to several limitations of the study, including the sample size limiting subgroup-analyses. Future studies will need to replicate and extend these findings with larger sample sizes. However, the proposed methodology in this proof-of-principle study provides a tool for the differentiation of distinct, biologically plausible subgroups.

## Footnotes

3 Hypnogogic-hypnopmpic experiences/occasional feeling that others look or talk about the subject.
