# Supplementary material for: Disaggregating physiological components of cortisol output: A novel approach to cortisol analysis in a clinical sample – A proof-of-principle study
Source: Neurobiol Stress. 2019 Mar 7;10:100153. doi: 10.1016/j.ynstr.2019.100153 (PMC6535687; doi:10.1016/j.ynstr.2019.100153)
Supplement: Multimedia component 1 [file mmc1.docx]

**Supplemental Information**

**Recruitment strategy and full inclusion and exclusion criteria, and pre-test screening on both days**

*Clinical cases Group (CG)*

Our clinical group was recruited from a cohort as part of an epidemiological study (PAATH: Prospective Analysis of At-risk-mental-states and Transitions into PsycHosis) run by the Cambridgeshire and Peterborough Early intervention team for psychosis (CAMEO) (1). The advantage of recruiting volunteers from the PAATh study was that patients had already undergone extensive diagnostic interviews and screening with regards to their mental health, medication, smoking and recreational drug use. The PAATh group consisted of help-seeking individuals, aged 16-35, referred to the service with a suspicion of a major mental disorder, indexed by emerging psychotic symptoms from February 2010 to September 2012 and who met criteria for High Risk mental states but not psychosis, according to the Comprehensive Assessment of At Risk Mental States (CAARMS) (2) at the time of assessment. They were followed up over a 2-year period.

The CAARMS identifies three At-risk groups (ARMS)

1. Attenuated Psychosis Group (AP):

Global Rating Scale Score of 3-5/6 on Unusual Thought Content subscale, 3-5/6 on Non-Bizarre Ideas subscale, 3-4/6 on Perceptual Abnormalities subscale, *or* 4-5/6 on Disorganised Speech subscales of the CAARMS, PLUS Frequency Scale Score of 3-6 on Unusual Thought Content, Non-Bizarre Ideas, Perceptual Abnormalities or Disorganised Speech subscales of the CAARMS for at least a week

1. Vulnerability Group (VA):

Family history of psychosis in first degree relative OR Schizotypal Personality Disorder in identified patient PLUS • 30% drop in Function score from premorbid level, sustained for a month, occurred within past 12 months OR score of 50 or less for past 12 months or longer

1. BLIPS

Global Rating Scale Score of 6 on Unusual Thought Content subscale, 6 on Non-Bizarre Ideas, 5 or 6 on Perceptual Abnormalities subscale or 6 on Disorganised Speech subscales of the CAARMS PLUS Frequency Scale Score of 4-6 on Unusual Thought Content, Non-Bizarre Ideas, Perceptual Abnormalities or Disorganised Speech subscales PLUS Each episode of symptoms is present for less than one week and symptoms spontaneously remit on every occasion PLUS Symptoms occurred during last year PLUS 30% drop in function score from premorbid level, sustained for a month, occurred within past 12 months OR Function score of 50 or less for past 12 months or longer

Participants between the ages 16-30 who had taken part in the PAATH trials were approached whether they would be willing to take part in a further study. A short telephone screen was conducted to ascertain whether participants were eligible for the study on the basis of our inclusion/ exclusion criteria. The participants took part in two full study days. Prior to consent they were made aware that they would undergo a stress induction. All participants had been diagnostically assessed by a psychiatrist in the team of JP and in addition MINI DSM-IV (3) diagnoses were obtained for all individuals.

Twenty-two ARMS participants were recruited. One participant was excluded because it emerged that he was unable to understand the instructions, one participant was excluded because she had become psychiatrically too unstable and had changed her medication from one session to the next. Twenty participants eventually completed the study. Diagnosis and Medication are summarized in Table S2.

### *Healthy Controls (HC)*

HC that had consented during the same period (February 2010-September 2012) to the PAATH trial were asked whether they were willing to take part in the Cortisol and Cognition study. The majority of the random sample of healthy volunteers (controls) for the PAATh trial had been recruited by using the postal address file (PAF) provided by Royal Mail. Recruiting HC from an existing trial cohort had the same advantages as for the CG. Three volunteers were recruited via poster advertisements in regional community colleges in Cambridgeshire and Peterborough. Twenty-two participants were recruited. One participant was not contactable after the first session and therefore excluded. Twenty-one participants completed the study.

*Inclusion and Exclusion criteria*

Participants were excluded if they were regular (daily) smokers, using recreational substances, regular alcohol use, high consumption of caffeinated drinks (equivalent of > 6cups of coffee), using regular steroid medication (asthma inhalers, steroid creams) were on antipsychotic medication, had made the transition to FEP, any chronic health problems (including high BP), or, for females, if they were on the combined contraceptive pill. Ideally participation of females should be during the second half of their menstrual cycle. However, initial screening suggested high prevalence of irregular or amenstrual cycles amongst participants in the patient group. In order to increase our chances of recruiting females into the study we compromised on this criterion.

Smoking status and recreational drug use was ascertained by accessing their records from the PAATh trial, in addition to the screening interview. Occasional/social smokers (<3day, not every day (i.e. evidence that able to stop) were included if they agreed to stop smoking for 5 days (2 days prior to collecting baseline CORT samples).

Participants were instructed not to consume alcohol on the night before, to abstain from caffeinated drinks in the morning and to not engage in vigorous physical activity including cycling to the sessions. They were offered re-imbursement for bus or taxi, which ever was least stressful for them.

On each study day they were asked to fill in questionnaires with regards to their medication status, smoking during the last month and last week, recreational drug/alcohol use and current medical illness on arrival. Records were checked for consistency. Any positive answer was explored further. We only went ahead with the tests if we were satisfied they still fulfilled our criteria (including a good night sleep) on the day of testing otherwise the session was re-arranged.

**Instruction for at home cortisol sampling, and procedure for in session cortisol sampling**

Participants were instructed to collect saliva samples using SalimetriCG ® collection kit (storage tube, swap, instructions and diary) straight after waking on two normal school/college/work days prior to attending the laboratory. The instructions were to keep sample tubes and a glass of water next to their bed, to rinse their mouth at waking and chew the swap until soaked (approximately 1-2min). They were also asked to avoid sample collection within 20 minutes of brushing teeth or a big dinner, 8 hours of consuming alcohol, 48 hours of smoking or consuming recreational drugs.

Hence, four sets of at home saliva were collected over the period of the study. Tubes were labeled and colour coded for day1/day2. In addition they were also instructed to note down the exact time of saliva collection and any missed samples in a diary provided by us, to keep the samples in a fridge after collection and hand them in on the day when they attended the laboratory.

In the laboratory we followed the same method of saliva sample collection. As the participants had already taken part in some tests in the morning they were offered some non-caffeinated drinks and low protein snack before the afternoon session. Prior to the first saliva sample collection they were asked to thoroughly rinse their mouth with water.

**Figure S1:** Testing protocol


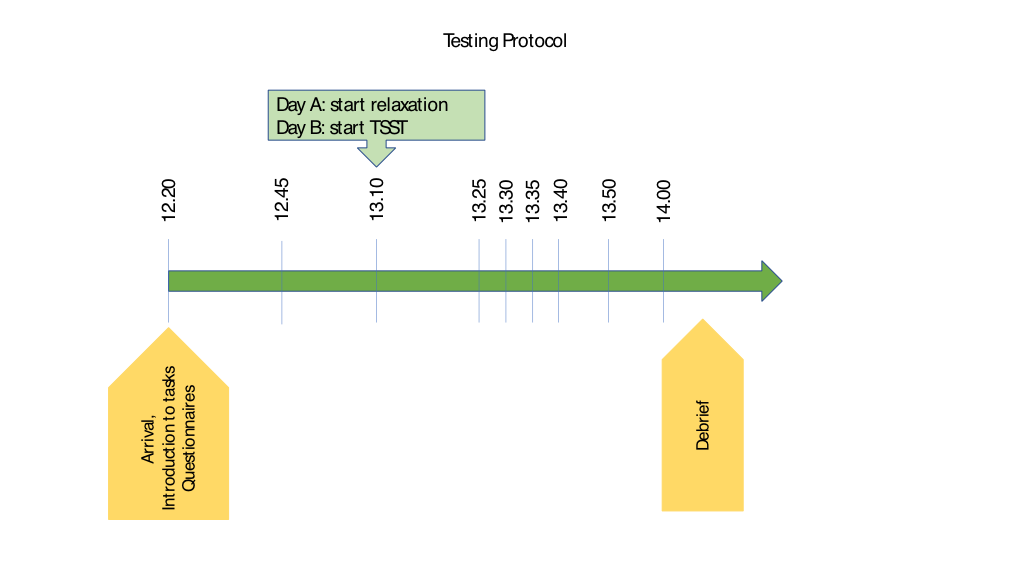


*Legend: Figure S1: Testing protocol:* Diurnal cortisol release changes at different rates throughout the day. In order to be able to control for background diurnal cortisol decline, participants were tested as close as possible to predetermined times on both testing days and throughout the study as illustrated below

**Table S1:** Times^a^ of collection of cortisol data, no extreme responders in sample

| Time- point | time of TSST data collection  mean (SD) | TSST minutes from start of task  mean (SD) | minutes between consecutive TSST time points  mean (SD) | Time of non-stress data collection  mean (SD) | “TSST minus non-stress cortisol” time difference mean (SD) |
| --- | --- | --- | --- | --- | --- |
| 2 ^b^ | 788 (2) | --- | --- | 787 (2) | 0 (3) |
| 3 | 806 (3) | 18 (2) | 18 (2) | No data collected | --- |
| 4 ^c^ | 811 (3) | 23 (1) | 5 (1) | 809 (2) | 2 (3) |
| 5 ^c^ | 816 (3) | 29 (2) | 5 (1) | 815 (2) | 1 (4) |
| 6 ^c^ | 823 (3) | 36 (2) | 7 (1) | 822 (2) | 1 (4) |
| 7 | 834 (4) | 46 (3) | 10 (2) | 833 (7) | 1 (8) |
| 8 | 845 (5) | 58 (5) | 12 (3) | No data collected | --- |

^b^ taken immediately prior to start of TSST

^c^ possible peak

**Figure S2:** Non-stress logged cortisol – imputed and raw in healthy controls and clinical group

*Legend Figure S2: non-stress raw and imputed scores by individual, separate by HC/CG:*

As some of the time periods when TSST cortisol was measured were very close together (eg. 5 minutes apart) and there was some individual variation in timing of the non-stress data points compared with the TSST data points (Table S1), we imputed non-stress logged cortisol by participant so that each individual had a non-stress cortisol value at the exact time when their TSST cortisol was assessed (all original non-stress values which were taken at the same time of day as TSST values were retained). As afternoon cortisol levels typically decline linearly, we imputed non-stress logged cortisol values using linear regression.

**Table S2: Primary and secondary diagnosis of patient group**

| **Participant (ARMS)** | **MINI**  **primary diagnosis** | **MINI SecondaryDiag1** | **MINI**  **SecondaryDiag2** | **Medication** |
| --- | --- | --- | --- | --- |
| 1 (AP) | major depressive episode recurrent | obsessive-compulsive disorder current |  | Citalopram, 20 mg |
| 2 (AP) | no primary current diagnosis (MINI) |  |  |  |
| 3 (AP) | No primary current diagnosis (MINI) | Psychotic disorders lifetime |  |  |
| 4 (BLIP) | major depressive episode recurrent | hypomanic episode past |  |  |
| 5 (AP) | generalized anxiety disorder current | major depressive episode past |  | Venlafaxine, 187.5 mg |
| 6 (AP) | generalized anxiety disorder current | major depressive episode recurrent |  |  |
| 7 (VG) | social phobia current non gen | major depressive episode past |  | Sertraline, 100mg |
| 8 (AP) | major depressive episode recurrent |  |  |  |
| 9 (AP) | major depressive episode recurrent | social phobia current gen |  |  |
| 10 (AP) | obsessive-compulsive disorder current | generalized anxiety disorder current | major depressive episode recurrent |  |
| 11 (AP) | major depressive episode recurrent | generalized anxiety disorder current |  | Citalopram, 20mg |
| 12 (AP) | no primary current diagnosis (MINI) |  |  | Medikinet XL, 30mg (used as needed, none 4 days prior to test) |
| 13 (AP) | major depressive disorder past |  |  | Citalopram, 30mg |
| 14 (AP) | generalized anxiety disorder current | major depressive episode recurrent |  |  |
| 15 (AP) | major depressive disorder current | agoraphobia | social phobia | Sertraline, 100mg |
| 16 (AP) | no primary current diagnosis (MINI) |  |  | Sertraline, 100mg |
| 17 (AP) | social phobia current gen | major depressive episode recurrent |  |  |
| 18 (AP) | no primary current diagnosis (MINI) |  |  |  |
| 19 (AP) | social phobia | major depression | agoraphobia |  |
| 20 (AP) | major depressive episode current |  |  | Citalopram, 40mg |

*Legend Table S2: Primary and secondary diagnosis of patient group:* All patients were referred because they were experiencing psychotic like symptoms. Most common main diagnosis was depression (n=8; recurrent=5, current=2, past=1) co-morbidity: OCD=1, hypomania=1, social phobia=1, generalized anxiety=1, agoraphobia=1). Three patients were diagnosed with Generalised Anxiety (n=3; co-morbidity: depression=3), three with Social Phobia (n=3; co-morbidity: depression=3) , one with OCD (n=1; co-morbidity: Generalised anxiety=1), one with a psychotic disorder (lifetime), four patients did not meet full criteria for any primary diagnosis according to the MINI diagnostic interview but showed sufficient impairment through their range of symptoms to justify follow up. First column contains participant number and At-risk mental state (ARMS) group: AP=attenuated psychotic symptoms; VG=Vulnerability Group; BLIPS

**Figure S3:** Extreme responders - TSST cortisol by group

*Legend Figure S3: Extreme Responders:* TSST cortisol change from start to peak was calculated (TSST cortisol peak minus baseline), with the box and whisker plot inspected for outliers. Three Clinical Group participants had extreme responses, with mean change scores being 6.5 times greater than the mean for the rest of the sample (M=0.88, SD=0.28 and M=0.14, SD=0.14 respectively). HC=healthy controls; CG=clinical group

**Table S3:** Slopes of “TSST minus non-stress cortisol” increase and decline phases and influence of primary predictors on these slopes

|  |  | **Cortisol slope increase** | | | | | | | | | | | | | | | | | **Cortisol slope decline** | | | | | | | | | | | | | | | |
| --- | --- | --- | --- | --- | --- | --- | --- | --- | --- | --- | --- | --- | --- | --- | --- | --- | --- | --- | --- | --- | --- | --- | --- | --- | --- | --- | --- | --- | --- | --- | --- | --- | --- | --- |
|  | **Confounds^a^** | | **n^b^** | | **Coef** | | **95% CI** | | | **P-value** | | |  | | | **Confounds** | | | | **n** | | | **Coef** | | | **95% CI** | | | **P-value** | | |  |  |  |
| **Main effects of time** | | | |  | |  | |  | | |  | | |  | | |  | | | |  | | |  | | |  | | |  | | |  |  |
| Time (linear) | N/A | | 136 (37) | | 2.59 x 10^-2^ | | 2.07 x 10^-2^ to 3.12 x 10^-2^ | | | <0.001 | | |  | | | N/A | | | | 155 (36) | | | -1.80 x 10^-2^ | | | -2.38 x 10^-2^ to -1.21 x 10^-2^ | | | <0.001 | | |  |  |  |
| Time2 (quadratic) | N/A | | 136 (37) | | 2.06 x 10^-4^ | | -1.77 x 10^-4^ 5.89 x 10^-4^ | | | 0.29 | | |  | | | N/A | | | | 155 (36) | | | -0.17 x 10^-4^ | | | -2.09 x 10^-4^ to 1.75 x 10^-4^ | | | 0.86 | | |  |  |  |
| **Primary predictor interactions with time** | | | | | | | | |  | | |  | | |  | | |  | | | |  | | |  | | |  | | |  | | |  |
| HC/CG*time | Gender x time  waking cortisol | | 132 (36) | | -10.57 x 10^-3^ | | -19.92 x 10^-3^ to -1.21 x 10^-3^ | | | 0.027 | | |  | | | Gender  Waking cortisol | | | | 151 (35) | | | 9.56 x 10^-3^ | | | 1.27 x 10^-3^ to 20.38 x 10^-3^ | | | 0.084 | | |  |  |  |
| CTQ*time | Gender x time  waking cortisol | | 127 (35) | | -0.09 x 10^-3^ | | -0.37 x 10^-3^ to 0.18 x 10^-3^ | | | 0.51 | | |  | | | Gender  Age x time  Waking cortisol | | | | 148 (34) | | | 0.31 x 10^-3^ | | | 0.03 x 10^-3^ to 0.59 x 10^-3^ | | | 0.028 | | |  |  |  |
| BDI*time | waking cortisol | | 132 (36) | | -0.41 x 10^-3^ | | -0.76 x 10^-3^ to -0.05 x 10^-3^ | | | 0.025 | | |  | | | Waking cortisol | | | | 151 (35) | | | 0.29 x 10^-3^ | | | -0.10 x 10^-3^ to 0.69 x 10^-3^ | | | 0.15 | | |  |  |  |

^a^ Putative confounders (IQ, age, test day order, gender, and waking cortisol) which correlated each predictor (group, CTQ, BDI) p<0.1 or r/ρr>=0.1 were also independently interacted with time on cortisol; those interactions which were p<.01 were included in each relevant model.

^b^ bracketed number refers to the number of cases. All TSST slope increase and decline models have 3 to 5 timepoints per person, which varies depending on when each individual peak occurred. Waking cortisol was missing for one HC participant, as was CTQ. The decline phase additionally excluded one participant whose cortisol declined from the start of TSST; cortisol decline was not calculated due to no prior increase

**Table S4:** Slopes of TSST cortisol increase and decline phases and influence of primary predictors on these slopes

|  | **Cortisol slope increase** | | | | | | | | | | | | | **Cortisol slope decline** | | | | | | | |
| --- | --- | --- | --- | --- | --- | --- | --- | --- | --- | --- | --- | --- | --- | --- | --- | --- | --- | --- | --- | --- | --- |
|  | **Confounds** | **n^a^** | | **Coef** | | **95% CI** | | **P** | | |  | | | **Confounds** | **n** | **Coef** | **95% CI** | **P** |  |  |  |
| **Main effects of time** | | |  | |  | |  | |  | | |  | |  |  |  |  |  | |  |  |
| Time (linear) | N/A | 136 (37) | | 2.09 x 10^-2^ | | 1.57 x 10^-2^ to 2.61 x 10^-2^ | | <0.001 | | |  | | | N/A | 155 (36) | -2.08 x 10^-2^ | -2.60 x 10^-2^ to -1.56 x 10^-2^ | <0.001 |  |  |  |
| Time2 (quadratic) | N/A | 136 (37) | | 1.50 x 10^-4^ | | -1.81 x 10^-4^ to 4.81 x 10^-4^ | | 0.37 | | |  | | | N/A | 155 (36) | -0.12 x 10^-4^ | -1.89 x 10^-4^ 1.65 x 10^-4^ | 0.90 |  |  |  |
| **Primary predictor interactions with time** | | | | | | | | | |  | | |  |  |  |  |  |  | | |  |
| HC/CG*time | Waking cortisol | 132 (36) | | -4.42 x 10^-3^ | | -14.47 x 10^-3^ to 5.62 x 10^-3^ | | 0.39 | | |  | | | Gender x time  Waking cortisol | 151 (35) | 11.45 x 10^-3^ | 1.84 x 10^-3^ to 21.06 x 10^-3^ | 0.020 |  |  |  |
| CTQ*time | Age x time  Waking cortisol | 127 (35) | | -0.07 x 10^-3^ | | -0.32 x 10^-3^ to 0.17 x 10^-3^ | | 0.56 | | |  | | | Gender x time  Waking cortisol | 148 (34) | 0.25 x 10^-3^ | 0.05 x 10^-3^ to 0.44 x 10^-3^ | 0.015 |  |  |  |
| BDI*time | Waking cortisol  Test day | 132 (36) | | 0.20 x 10^-3^ | | -0.56 x 10^-3^ to 0.16 x 10^-3^ | | 0.27 | | |  | | | Waking cortisol | 151 (35) | 0.35 x 10^-3^ | 0.05 x 10^-3^ to 0.65 x 10^-3^ | 0.023 |  |  |  |

^a^ bracketed number refers to the number of cases. All TSST slope increase and decline models have 3 to 5 timepoints per person, which varies depending on when each individual peak occurred. Waking cortisol was missing for one HC participant, as was CTQ. The decline phase additionally excluded one participant whose cortisol declined from the start of TSST; cortisol decline was not calculated due to no prior increase.

**References**

1. Hui C, Morcillo C, Russo DA, Stochl J, Shelley GF, Painter M, *et al.* (2013): Psychiatric morbidity, functioning and quality of life in young people at clinical high risk for psychosis. *Schizophr Res*, 2013/06/19. 148: 175–180.

2. Yung AR, Yuen HP, Phillips LJ, Francey S, McGorry PD (2003): Mapping the onset of psychosis: The comprehensive assessment of at risk mental states (CAARMS). *Schizophr Res*. 60: 30–31.

3. Sheehan D V., Lecrubier Y, Sheehan KH, Amorim P, Janavs J, Weiller E, *et al.* (1998): The Mini-International Neuropsychiatric Interview (M.I.N.I.): The development and validation of a structured diagnostic psychiatric interview for DSM-IV and ICD-10. *J Clin Psychiatry*. (Vol. 59), pp 22–33.
